# Bilateral Ovarian Clear Cell Carcinoma Arising in 17 Year Longstanding History of Bilateral Ovarian Endometriosis

**Published:** 2017-01-01

**Authors:** Thin Thin Win, Nik Mohamed Zaki Nik Mahmood, Saung Oo Ma, Mazita Ismail

**Affiliations:** 1 *Dept. of Pathology, School of Medical Sciences, Universiti Sains Malaysia, 16150 Kubang Kerian, Kelantan, Malaysia*; 2 *Dept. of Obstetrics and Gynaecology, School of Medical Sciences, Universiti Sains Malaysia,, 16150 Kubang Kerian, Kelantan, Malaysia*; 3 *Dept. of Pathology, Hospital Raja Permaisuri Bainun, 30990 Ipoh, Perak, Malaysia*

**Keywords:** Clear Cell Carcinoma, Ovarian Endometriosis, Malignant Transformation

## Abstract

Clear cell carcinoma of ovary is uncommon ovarian tumour that arises from surface epithelium of ovary. It has well-known association with ovarian endometriosis. We report here the first case of bilateral clear cell carcinoma of ovaries in a 40-year-old woman with a 17-year history of bilateral ovarian endometriosis. In addition, during the longstanding duration of the endometriosis, the patient was treated with hormonal therapy, including oestrogen. It represents the first report of such bilateral involvement in the background of ovarian endometriosis. This should prompt clinicians to be aware that prolonged hormonal treatment of endometriosis may precipitate bilateral malignancy of the ovary.

## Introduction

Clear cell carcinoma (CCC) of the ovary is an uncommon malignant ovarian tumour that arises from surface epithelium of ovary. Although it can occur bilaterally or unilaterally, the incidence of bilaterality is less than 10% (1), is found throughout the stages of cancer development. The prevalence of bilateral involvement found in stage I was reported as 4% (2).

30%-35% of reported ovarian CCC cases are associated with endometriosis either in the involved ovary or elsewhere in the pelvis or abdomen (2). Although malignant transformation of endometriosis is infrequent, there are some reported cases in both gonadal and extragonadal sites (3, 4). Among them, endometrioid adenocarcinoma is the most common, followed by CCC (3).

Bilateral ovarian CCC is present in rare prevalence and they have never been reported with the background of bilateral endometriosis. Here we report a case of bilateral CCC of the ovaries with 17 year underlying history of bilateral ovarian endometriosis. To the best of our knowledge, it represents the first report of bilateral ovarian CCC with a long history of underlying bilateral endometriosis in a middle-aged woman.

## Case report

A 40-year-old woman, parity 1+1 complained of worsening dysmenorrhoea and palpable abdominal mass long diagnosed as endometriosis. The diagnosis of the endometriosis was established 17 years ago. 

At the onset of the first complaint 17 years ago, she was presented with abdominal emergency, followed by laparotomy. Bleeding from right fimbrial cyst in Pouch of Douglas (POD) was noted. Histopathological finding was consistent with endometriosis. She was treated with danazol and hormonal therapy (combined estrogen and progestin **o**ral contraceptive pills). She was noted for primary infertility, conceived 6 yr later and delivered a live baby by caesarean section. She had presented again 2 yr later for dysmenorrhoea and left ovarian cyst was noted on ultrasound examination. Although patient was planned for surgical removal of the ovarian cyst, operation defaulted with irregular follow-up.

On the most current physical examination, a huge mass was palpable in pelvis, extending towards the lower abdomen. It was 180 x 150 mm in size with irregular border, cystic to firm in consistency and fixed. There was no ascites and no hepatosplenomegaly. Vaginal and speculum examination revealed normal healthy cervix. However, the POD was full with mass. CT scan and intravenous pyelogram (IVP) revealed bilateral hydronephrosis, left ovarian tumour and endometriosis of the right ovary. Tumour marker study revealed CA-125 level was 1424 U/ml (Reference range: <35U/ml). Patient was then planned for exploratory laparotomy attended by gynecological, surgical and urology teams. Informed consent was taken from the patient. 

At the time of the exploratory laparotomy, the whole lower abdomen was filled with bilateral ovarian masses, slightly larger on the left. Right ovarian mass was cystic with intact capsule. Left ovarian mass was partly cystic, partly solid and was ruptured at the cystic part with chocolate materials. Uterus was bulky and POD was filled with dense adhesion and serous fluid. Multiple enlarged lymph nodes were seen in the pelvis. Ureter stenting was done, followed by total abdominal hysterectomy, bilateral salpingo-oophorectomy and omentectomy. 

**Fig. 1:**
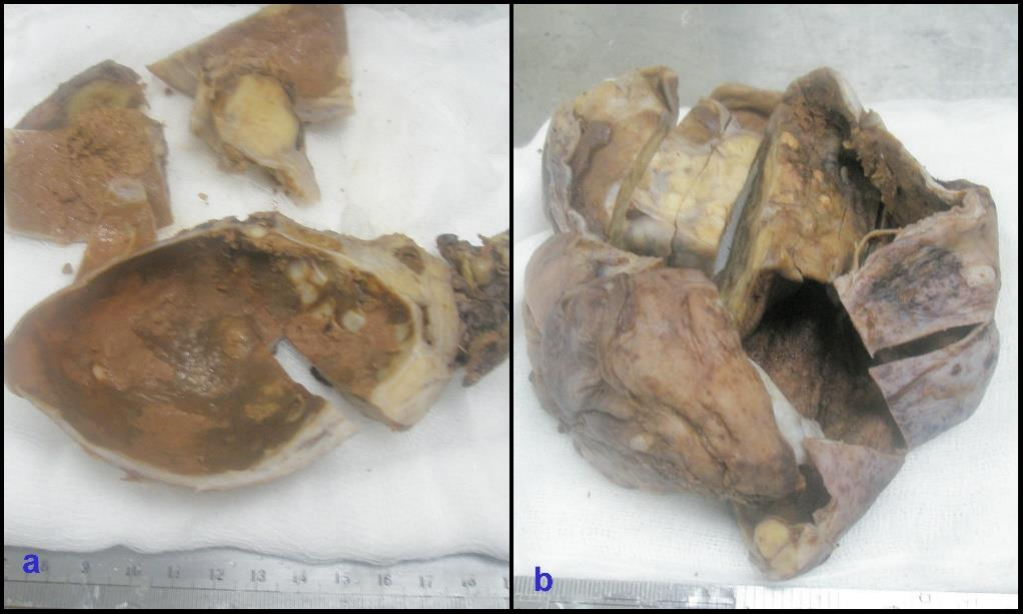
a) Right ovarian tumor which is mainly cystic and partly solid at the periphery. (b) Left ovarian tumor which is partly solid and partly cystic; Both cystic walls are lined by old hemorrhagic areas

 On gross examination of surgical specimen, uterus was slightly enlarged and cut surface showed intramuscular grayish white mass with whirling pattern measuring 60 mm in diameter. Cervix was healthy. Right, ovarian mass measured 35×80×35mm, lobulated with intact capsule. Cut sections showed two loculated cysts filled with brownish black material. Solid grayish white area with circumscribed mass measuring 25mm in diameter was seen adjacent to cystic spaces (Fig. 1a). Left ovarian mass measured 150×130×35mm, lobulated and cut sections showed partly cystic and partly solid (Fig. 1b). Cystic spaces were 120mm in diameter and solid area measured 115mm in diameter. Part of cystic wall was ruptured with chocolate colored material. Serial cut sections of solid area revealed foci of calcification and hemorrhage with soft to firm areas. Cystic spaces contained greenish jelly-like materials and inner lining of cysts was nodular.

**Fig. 2:**
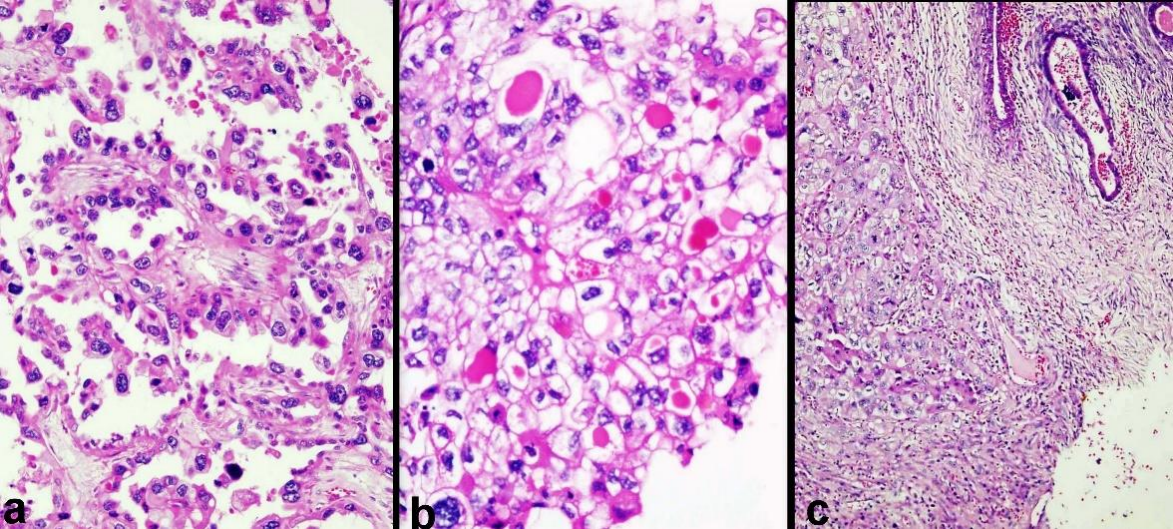
H & E stain (a) Clear cell carcinoma with papillary configuration lined by pleomorphic hobnail cells (X200); (b) Clear cell carcinoma showed large cells with clear abundant cytoplasm and some of the cytoplasm of the cell containing eosinophilic hyaline bodies (X400); (c) Foci of endometriosis in close proximity with clear cell carcinoma of right ovary (X100

 On histopathological examination, intrauterine mass revealed leiomyoma. Endometrium was in proliferative phase. Cervix was unremarkable. Cystic part of both ovarian masses revealed cystic walls lined by normal looking endometrial lining with endometrial stroma and hemosiderin-laden macrophages. Solid areas of the both ovarian masses revealed infiltrating tumours composed of diffuse sheaths of large pleomorphic cells with abundant clear cytoplasm. Tumour cells were forming solid sheets, glandular and papillary pattern (Fig. 2a). Most of pleomorphic cells were polygonal and have hyperchromatic to vesicular nuclei and prominent nucleoi. Some of the tumour cells have vacuolated cytoplasm, some have pink granular oxypholic cytoplasm and some showed hob-nailing pattern. Mitotic activities were abundant. In some of glands and tubules, pink intra-cytoplasmic hyaline bodies were seen (Fig. 2b). Some parts of the cystic areas were also lined by tumour cells. Omentum showed no tumour deposits. Sections from right ovary showed foci of endometrial glands in close proximity to foci of clear cell carcinoma (Fig. 2c). Sections were taken from ruptured area of left ovarian mass also showed cyst wall with endometrial lining and no foci of CCC was seen in the cyst lining. Pelvic lymph nodes also showed metastatic clear cells of same morphology. With above histological findings, bilateral CCC in underlying bilateral endometriosis of both ovaries was diagnosed with FIGO (The International Federation of Gynaecological Oncologists) stage IIIC.

After exploratory laparotomy and removal of bilateral ovarian masses, CA-125 level reduced to 403 U/ml. 6 cycles of chemotherapy, combined Cisplatin and paclitaxel (TAXOL^®^) was given. After completion of chemotherapy, clinical condition of the patient was worsened with developing pleural effusion and ascites. Recheck CT scan revealed multiple lung metastatic nodules with pleural effusion, metastatic liver nodules and multiple enlarged pelvic lymph nodes with ascites. CT scan of the brain also revealed multiple metastatic nodules. Patient succumbed to disease 10 months after surgery.

## Discussion

We have reported for the first time a case with bilateral involvement of ovarian CCC where bilateral ovarian endometriosis was shown as the underlying pathology. CCC has a well-known association with both gonadal and extragonadal endometriosis. Twenty percent of ovarian clear cell carcinoma is malignant tumours arising from underlying ovarian endometriosis (5).

Unopposed oestrogen stimulation in endometriosis is one of the factors for malignant transformation of endometriosis (6). Our patient had a long-history of endometriosis treated with hormonal therapy (combined estrogen and progestin **o**ral contraceptive pills) for more than 10 yr. This may explain the malignant transformation of the ovarian endometriosis, although bilateral involvement of CCC has not been reported before. Moreover, such a long-standing history of endometriosis was not seen in the previous literature. 

Patients with ovarian CCC are usually in the fifth or sixth decades, although, a case of clear cell carcinoma was reported in a 19-year-old girl (7). Our patient showed earlier onset as compared to the usual cases. Patients with CCC arising from endometriosis are generally younger than those with general CCC are (8, 9). Our case was found in stage I (b), with no evidence of capsular invasion by tumour and no evidence of pelvic and omental spread. Foci of endometriosis are seen in close proximity to CCC or CCC may arise within endometriosis (2). In this case, endometrial glands were also seen in close proximity to CCC.

Bilateral involvement of the ovarian CCC may be due to the metastasis from either the kidney or the contralateral ovary. In this report, we provide evidence that excludes such possibility. Both CT and intravenous IVP showed there was no renal tumor mass apart from bilateral hydronephrosis due to pressure effect of bilateral ovarian mass on ureters. Histological sections taken from rupture area of left ovarian mas showed only endometriotic cyst and no features of CCC. Omentum and ascetic fluid cytology showed no tumour cells. Therefore, we ascertained that bilateral involvement of CCC was not due to metastatic involvement from contralateral ovary to another; and they developed as primary malignancies from each ovary. 

CCC is the most common epithelial ovarian neoplasm to be associated with paraneoplastic hypercalcemia, although exact mechanism is unknown (2). Two percent of ovarian CCC was associated with hypercalcemia (10). In our case, serum calcium was within normal limit.

Poor prognostic factors in ovarian CCC include young age (<60 yr), advanced stage of disease and the presence of vascular invasion (11). Histological grade is one of the significant prognostic factors in patients with CCC of the ovary (12). This case was poor in prognosis with stage IIIC, high-grade nuclear features and bilateral involvement of both ovaries. Therefore, extensive distant metastasis of tumour developed even after complete chemotherapy.

## Conclusion

We reported a unique case of bilateral ovarian CCC in a middle-aged woman that prompt clinicians to be aware that prolonged hormonal treatment of endometriosis may precipitate bilateral malignancy of the ovary.

## Conflict of Interests

The authors declare that there is no Conflict of Interests. 
